# Impact of Genitourinary Injuries on Patients Requiring an Emergency Laparotomy for Trauma

**DOI:** 10.7759/cureus.6826

**Published:** 2020-01-31

**Authors:** W. Britt Zimmerman, Alfred E Baylor, Lisa Hall Zimmerman, Heather Dolman, Jeremy R Ciullo, Jessica Dornbush, Andrew R Isaacson, Roozbeh Mansour, Robert F Wilson, James G Tyburski

**Affiliations:** 1 Urology, Michigan State University, East Lansing, USA; 2 The Michael and Marian Ilitch Department of Surgery, Wayne State University School of Medicine, Detroit Receiving Hospital, Detroit, USA; 3 Department of Pharmaceutical Services, Beaumont Health, Royal Oak, USA

**Keywords:** penetrating, trauma, genitourinary, urologic, mortality, urology, acute care surgery

## Abstract

Introduction

In patients having emergency abdominal surgery for trauma, the presence of urologic injury tends to increase mortality and morbidity.

Methods

This retrospective study evaluated patients requiring emergency surgery for abdominal trauma at a Level 1 Trauma Center over 30 years (1980-2010). Special attention was given to patients with concomitant genitourinary (GU) injuries.

Results

Of 1105 patients requiring an emergency laparotomy for trauma, 242 (22%) had urologic injuries including kidney 178 (16%), ureter 47 (4%), and bladder 46 (4%). Of the 242 patients, 50 (20%) died early (<48 hours) and 13 (5%) died later, primarily due to infection. A concept of “seven deadly signs” of hypoperfusion was developed. In patients with GU injuries, the presence of any deadly sign of hypoperfusion increased the mortality rate from 4% (6/152) to 63% (56/90), p<0.001. Of the 53 patients having a nephrectomy, 36 (68%) had one or more deadly signs and 27 (75%) died. Of 17 without deadly signs, only 2 (12%) died (p=0.001). Of 167 GU patients receiving blood, 59 (35%) developed infection vs 3/75(4%) in those receiving no blood (p<0.001).

Conclusions

The presence of deadly signs of severe injury and hypoperfusion on admission was the major factor determining mortality. With a severely injured kidney plus any deadly signs of hypoperfusion, special efforts should be made to avoid a nephrectomy.

## Introduction

Trauma is the leading cause of death in the United States in patients aged 46 years or younger [1]. In the literature, genitourinary (GU) injuries are found in 10-40% of patients presenting with abdominal trauma requiring emergency surgery [2-3]. In previous studies, the incidence of each of the urological injuries associated with abdominal trauma has included kidney (10-47.8%), ureter (1-2%), and bladder (1-2%) [2,4].

In some reports of GU trauma, the injuries are isolated, making management easier and prognosis better; however, this seldom occurs in reported series and guidelines [2,4]. The objective of this study was to evaluate the factors associated with mortality and morbidity in 242 patients with GU injuries in a group of 1105 patients with abdominal trauma requiring emergency laparotomy.

## Materials and methods

This retrospective, institutional review board-approved cohort study evaluated consecutive patients who were admitted with abdominal trauma requiring emergency surgery at an urban, Level 1 Trauma Center, over 30 years (1980-2010). The records of patients who had concomitant GU injuries, including bladder, ureter, and/or kidney, were of primary interest.

Baseline data included age, severity of injury, concomitant injuries, emergency department (ED), operating room (OR), and laboratory data were also evaluated. Mortality was stratified into early deaths (less than 48 hours) and late deaths (48 hours or more after admission). 

Transfusions evaluated included packed red blood cells (pRBCs) given in the first 24 hours following admission to the emergency department. Massive transfusion was defined as ten or more units of pRBC given in the first 24 hours. Infections evaluated included pneumonia (PNA), bacteremia (BSI), intraabdominal abscesses/peritonitis (IAI), urinary tract (UTI), or surgical wound (SWI).

Statistical analyzes were performed using SPSS v21.0. Univariate analyses evaluated baseline differences between groups. Categorical variables were compared using Pearson’s χ2 analysis. Continuous variables were analyzed with the Student’s t-test or Mann-Whitney U test as appropriate. Statistical significance for analyses was defined as a p-value < 0.05.

## Results

Incidence

Of the 1105 patients presenting with abdominal trauma requiring emergency surgery, 242 (22%) had one or more GU injuries, including bladder in 46 (4%), ureter in 47 (4%), and kidney in 178 (16%). The overall mean age was 32±13 years with a mean Injury Severity Score (ISS) of 17±10 (Table 1). For the GU injuries, penetrating trauma (hand gun, shotgun, and stab wounds) was present in 210 (90%) with the remaining (10%) sustaining blunt trauma. Overall, the GU injury patients had a mean higher ISS (21±11 vs 16±10, p<0.001) and a higher mortality rate (26%, 62/242 vs 16%, 138/863, p<0.001) compared to patients with no GU injury. In the GU trauma patients, the kidney injuries had the highest mean ISS (23±11) and highest mortality rate (31%, 55/178). Bladder injuries had the lowest ISS (16±9) and the lowest mortality rate (13%, 6/46).

**Table 1:** Baseline characteristics GU: Genitourinary

Characteristics	All N=1105	No GU Injury N=863	GU Injury N=242	P-value (GU vs No GU)
Age, years*	32± 13	32±13	31±12	0.06
Injury Severity Score	17±10	16±10	21±11	<0.001
Mortality rate	200/1105 (18%)	138/863 (16%)	62/242 (26%)	0.001
Penetrating trauma type	911 (82)	701 (81)	210 (87)	0.04
Genitourinary injuries				
Kidney	178	-	-	-
Ureter	47	-	-	-
Bladder	46	-	-	-

The causes of penetrating GU trauma included hand gun wounds (188, 78%), shotgun (SGW) (17, 7%), and stabs (12, 5%). Blunt trauma included motor vehicle collision (MVC, 19, 8%) and assault or fall (6, 2%). The most lethal of the various causes of urologic trauma were 47% for MVC (9/19) and 47% for SGW (8/17). The mortality rate for the remaining patients was 25% (3/16) for stabs, 24% (46/188) for hand gun wounds, and 17% (1/6) for falls or assault. The 188 hand gun wounds involved bladder in 37 (20%), ureter in 41 (22%), and kidney in 131 (70%). In 17 SGW patients, 4 had bladder and 13 had kidney without any ureter involvement. The 12 stab wounds, 1 had bladder and 11 had kidney. Of the 145 firearm injuries to the kidney (136 hand gun wounds and 12 SGWs), 63 (44%) caused severe renal injuries (Grade IV/V). Of the 18 motor vehicle collisions causing kidney injury, 15 (83%) caused severe kidney injuries, p<0.005. The 25 blunt trauma patients had 29 GU injuries including 23 kidney, 4 bladder, and 2 ureter injuries. 

The non-urologic injuries associated with the highest incidence of GU trauma included the spleen (65/156, 42%), pancreas (114/336, 34%), rectum (19/70, 27%), and liver (82/302, 27%). Kidney injury was most likely to be associated with spleen (63/156, 40%), pancreas (109/336, 32%), and vascular injuries (51/291, 18%). Surgical intervention for patients with a kidney injury included surgical packing in 36/178 (20%), repair in 89/178 (50%), or nephrectomy in 53/178 (30%).

Of the 863 associated injuries in the 242 patients with GU trauma (3.6 per patient), the most frequent were colon 132/242 (55%), stomach 127/242 (52%), pancreas 114/242 (47%), and small bowel 107/242 (44%). Other associated injuries were liver 84 (35%), chest 69 (29%), vascular 67 (28%), and spleen 64 (26%). Although pancreas was the most frequently associated injury with kidney trauma (109/178, 61%), it was very uncommon with isolated bladder (1/38, 3%) and isolated ureter (4/23, 17%).

The associated injuries with the highest mortality rates overall were vascular 38/67 (57%), head (central nervous system (CNS)) 10/21 (48%), chest 29/69 (42%), liver 33/84 (39%), duodenum 17/45 (38%), spleen 23/64 (36%), and pancreas 32/114 (28%). The associated injuries with the highest infection rates included spine 8/10 (80%), head (CNS) 8/11 (73%), abdominal vascular injuries 19/31 (61%), pancreas 42/82 (51%), and duodenum 14/28 (50%). Infection rates with other associated injuries were spleen 19/41 (46%), chest 17/39 (44%), colon 39/101 (39%), and liver 18/54 (35%).

Deadly signs

There were “seven deadly signs” of hypoperfusion or severe injury associated with a greatly increased mortality rate. These seven deadly signs included pRBC transfusions of 20 or more units, selected severe vascular injury (aorta, inferior vena cava (IVC)), superior mesenteric artery (SMA), or portal vein), Revised Trauma Score 0-4.9, ED systolic blood pressure (BP) < 50 mmHg, initial OR systolic BP < 70 mmHg, arterial to end tidal pCO2 difference (P(a-ET) CO2) of greater than 18 mmHg, lowest OR temperature less than 33°C, and last OR temperature less than 34°C. The deadly signs correlated significantly with ISS and mortality (Table 2). All of the patents with one or more deadly signs had significantly higher ISS scores than patients with no deadly signs (25±9 versus 14±8, p<0.001).

**Table 2:** Seven deadly signs correlated with ISS and mortality rate ^number of patients (%); *p<0.001 compared to those with deadly signs; GU: Genitourinary; ISS: Injury Severity Score; pRBC: packed red blood cells; IVC: Inferior Vena Cava; SMA: Superior Vena Cava; ED: Emergency Department; BP: Blood Pressure; OR: Operating Room; (P(a-ET) CO2): Arterial to End Tidal CO2 Difference

Seven Deadly Signs	ISS N=1105	Mortality Rate^ No GU Injury N=863	Mortality Rate^ GU Injury N=242
≥20 units pRBC	27±11	68/94 (72%)	32/49 (65%)
Vascular injury (aorta, IVC, SMA, or portal vein)	25±11	67/132 (51%)	31/45 (69%)
Revised Trauma Score 0-4.9	27±13	69/91 (76%)	26/29 (90%)
ED systolic BP < 50 mmHg	28±13	52/72 (72%)	23/27 (85%)
Initial OR systolic BP < 70 mmHg	26±11	49/57 (86%)	19/22 (86%)
(P(a-ET) CO_2_) > 18 mmHg	24±9	20/31 (65%)	5/10 (50%)
OR temp, lowest < 33 or final < 34, °C	24±9	24/45 (53%)	9/11 (82%)
No deadly signs	14±8*	15/628 (2%)*	6/152 (4%)*
Any deadly sign	22±10	123/235 (52%)	56/90 (63%)

The incidence of deadly signs was higher with GU injuries (63%, 152/242) than that in patients without GU injuries (27%, 235/863, p<0.001). (Table 2). If one or more deadly signs were present, the patients with GU injuries tended to have higher mortality rates than those with non-GU injuries (63% (56/90) vs 52% (123/235), p=0.10). With three or more deadly signs, patients with GU injuries had a mortality rate of 91% (50/63). (Table 3)

**Table 3:** Number of deadly signs present correlated with ISS and mortality rate GU: Genitourinary; ISS: Injury Severity Score; ^number of patients (%); *p<0.001 compared to those with deadly signs

Number of Deadly Signs Present	ISS N=1105	Mortality Rate^ No GU injury N=863	Mortality Rate^ GU Injury N=242	Mortality Rate with Nephrectomy	Nephrectomy N=53	No Nephrecotomy N=125	Grade IV Renal Injury N=67	Grade V Renal injury N=37
None, n=780	14±8*	15/628 (2%)*	6/152 (4%)*	2/17 (12%)	17 (32%)	86 (69%)	33 (49%)	9 (24%)
1, n=131	22±10	23/94 (24%)	12/37 (32%)	8/16 (50%)	16 (30%)	13 (10%)	11 (16%)	12 (32%)
2, n=76	25±9	30/56 (54%)	14/20 (70%)	5/5 (100%)	5 (9%)	12 (10%)	12 (18%)	2 (5%)
≥ 3, n=118	28±12	70/85 (82%)	30/33 (91%)	14/15 (93%)	15 (28%)	14 (11%)	11 (16%)	14 (38%)

Mortality

The traumas with the highest mortality rate were MVC (9/19, 47%), SGW (8/17, 47%), and handgun wounds (41/188, 21%). Lower mortality rates were seen with stabs (3/12, 25%), hand gun wounds (45/199, 24%), and falls or assault (1/6, 17%). Mortality was highest in the patients with Grade IV/V (n=104) renal injuries, 50/104 (48%) versus 7/74 (9%) with Grade I-III (n=74), p<0.001. The primary organs injured related to death included vascular injury (60%), liver (53%), pancreas (52%), chest (48%), and small bowel (48%). For the 13 patients who died after 48 hours, the factors that seemed most important in their deaths included nephrectomy in eight (62%, p=0.002) patients, massive transfusion in eight (62%, p=0.04) patients, and infections in seven (54%, p=0.02) patients (Table 4).

**Table 4:** Grade of renal injuries associated with mortality, infection and length of stay ISS: Injury Severity Score; pRBC: packed red blood cells; LOS: length of stay; ^mean±SD, *N(%), #in patients surviving > 48 hours

Grade of Renal Injury	Patient No. (Mortality Rate)	Nephrectomy	ISS^	Infection*	pRBC>20 units	LOS^#
I	10 (0%)	0	9±6	2 (4%)	0 (0%)	15±10
II	25 (0%)	0	12±5	7 (14%)	1 (2%)	15±14
III	39 (18%)	0	18±5	15 (30%)	7 (17%)	22±25
IV	67 (39%)	20 (30%)	24±6	19 (38%)	15 (36%)	24±22
V	37 (65%)	33 (89%)	34±13	7 (14%)	19 (45%)	16±15
Total Patients	178	53	23±11	50	42	21±20

When 104 patients with severe kidney injuries (Grade IV and V) were examined for the effect of nephrectomy on mortality rates, it appeared as if nephrectomy itself greatly increased mortality (29/53 (55%) vs 26/125 (21%), p=0.001. (Table 5) However, when the results were considered in terms of the grade of renal injury, the results appeared different (Table 4). Nephrectomy patients were more likely to have deadly signs (36/53, 68% vs 39/125, 31%, p=0.001). If the kidney was injured, but not removed, infections were more common, [32% (40/125) vs 19% (10/53), (p=0.04)]. When patients with an isolated GU injury (involving only one GU organ) were evaluated, a higher mortality rate was identified with isolated kidney (29%, 45/153) and isolated ureter (17%, 4/23) injuries compared to isolated bladder injuries (8%,3/38, p<0.05). In addition, LOS was longer in patients whose only GU injury was kidney compared to bladder injuries, (20±21 vs 14±16, days, p<0.05).

**Table 5:** Impact of nephrectomy in patients with renal injuries ^number of patients (%); ISS: Injury Severity Score; LOS: Length of Stay

	Nephrectomy N=53	NO nephrectomy N=125	P-value
Overall ISS, mean±SD	28±12	19±9	<0.001
Overall Mortality Rate^	29/53 (55%)	26/125 (21%)	<0.001
Mortality Rate with Deadly Signs	27/36 (75%)	24/39 (62%)	0.31
Mortality Rate without Deadly Signs	2/17 (12%)	2/86 (2%)	0.12
Incidence of Deadly signs	36/53 (68%)	39/125 (31%)	0.001
Infections in pt surviving > 48 hr^	10/53 (19%)	40/125 (32%)	0.04
LOS, in pt surviving to discharge, mean±SD	16±17	21±21	0.09

If the ISS values were stratified into low (4-15), moderate (16-24), and high (25-75) levels, there were no deaths in the 164 GU injury patients with a low ISS. In the 67 GU patients with an ISS of 16-24, the mortality rate was 16% (11/67) and in patients with a high ISS (25 or more), the mortality rate was 46% (51/111) (p<0.001). Grade IV/V renal injuries were more likely to receive 10 or more units of pRBC (54/104 (52%) vs 16/74 (35%), p<0.001) and have an OR BP < 70 mmHg (16/104 (15%) vs 3/74 (4%), p=0.02) versus patients with Grade I-III renal injury.

Other factors affecting the mortality rate included blood transfusions and age. In patients with GU injury, mortality progressively increased from 4% with no blood transfusions up to 65% in those who received 20 or more units of pRBC (Table 6). Of the 49 GU injury patients who were 40 years of age or more, 29 (59%) died. This was significantly higher than the 22% (42/193) mortality rate in younger patients, p<0.001. The incidence of deadly signs was higher in patients ≥ 40 years of age, (51% vs 34%, p=0.025), as was the mean ISS (26±12 vs 20±10, p=0.005). The infection rates were similar, but the length of stay (LOS) tended to be longer in patients aged ≥ 40 years (22±22 vs 18±19, days, p=0.42).

**Table 6:** Blood transfusions correlated with mortality and infection rates ^number of patients (%); *p<0.001 compared to pRBC-none; #p=0.005 compared to pRBC-none; ⱡp=0.006 compared to pRBC-none; pRBCs: packed red blood cells; +in patients surviving > 48 hours

Number of Blood Transfusions^	All Patients N=1105	Mortality Rate No GU injury N=863	Mortality Rate GU Injury N=242	Infection Rate^+^ No GU injury N=740	Infection rate GU Injury N=192
pRBC – none	510	15/435 (3%)	3/75 (4%)	61/414 (15%)	10/72 (14%)
pRBC – 1-9 units	335	22/253 (9%)#	16/82 (20%)ⱡ	89/237 (37%)*	26/71 (37%)*
pRBC – 10-19 units	117	33/81 (41%)*	11/36 (31%)*	28/55 (51%)*	17/29 (59%)*
pRBC – ≥20 units	143	68/94 (72%)*	32/49 (65%)*	24/34 (71%)*	10/20 (50%)*

In light of the management of critically ill patients evolving over the last 30 years, evaluation of mortality associated with severity of injury was compared. The following three time periods were compared: 1980-1989 patients’ ISS was 22.8±9.3 with a mortality rate of 13/54 (24%), 1990-1999 patients’ ISS was 19.5±9.2 with a mortality rate of 30/118 (25%), and 2000-2010 patents’ ISS was 21.5±2.9 with a mortality rate of 19/70 (27%).

Infections

Overall, there were 932 patients who survived > 48 hours in which infections were evaluated. Of the 192 patients with GU injury surviving more than 48 hours, infections developed in 63 (33%) compared to the 202/740 (27%) non-GU injury patients with infections, p=0.13. In the GU injury patient, the most common infection was pneumonia (41/63, 65%) followed by intra-abdominal abscess and/or peritonitis (33/63, 52%). The most significant factor associated with the development of infection was the administration of one or more units of blood (54/167, 32% blood vs 10/75 (13%) no blood (p=0.002). Of the 192 GU injury patients living greater than 48 hours, the infection rate increased from 4% (3/75) for no blood transfusions to 20% (16/82) for 1-9 units, 31% (11/36) for 10-19 units, and 65% (32/49) for 20 or more units, p<0.001 (Table 6). The infection rates were also more common with blunt trauma 10/15 (67%) vs 54/165 (33%) for penetrating trauma (p<0.01). In addition, mortality rates after 48 hours were higher in those who developed infection (11% vs 5%, p=0.01).

Length of Stay

For the patients discharged home alive, GU injury was associated with a longer LOS, 19±20 vs 15±17 days in those without GU injury (p=0.003). In addition, the LOS in patients with kidney injuries (20±21 days) and ureter (20±22 days) were longer than in those with bladder injuries (15±15 days, p<0.05). Other factors causing a prolonged LOS included an ISS > 25 (28±27), presence of deadly signs (28±28), and blood transfusions (23±23 days, p<0.001).

## Discussion

This is one of the largest series of penetrating urologic injuries, especially hand gun wounds of the kidney, being reported. Traumatic injuries requiring emergency laparotomy are associated with significant morbidity and mortality. In the majority of abdominal trauma patients, mortality is primarily associated with the severity of the injuries, hypoperfusion, and the amount of blood loss. Concomitant genitourinary (GU) injury, especially to the kidney, occurs in up to 40% of patients with abdominal trauma [2-3, 5]. When compared to our abdominal trauma patients without GU injuries, those with GU trauma had a higher severity of injury (ISS 21±11 vs 16±10) and higher mortality rates (26% vs 16%, p<0.001). All 242 patients in this series had another abdominal injury. In most series, at least 95% of bladder, ureter, or kidney are associated with other abdominal organ injuries [5-6].

As noted in large retrospective series, renal trauma is seen primarily in patients aged 20-30 years [5]. It is also known that 82-95% of renal injuries reported in the US are due to blunt trauma; however, 90% of our renal injury patients had penetrating trauma, of which 94% were due to firearms (GSW and SGW) [5]. The literature reports that renal trauma associated with penetrating trauma is more likely to require surgical intervention [5]. Trauma usually has to be quite severe to cause renal injuries and at least 2 or 3 abdominal organ injuries are usually also present [5]. When patients with abdominal trauma are hemodynamically unstable, they are much more likely to be explored and if a kidney injury is the cause, it is more apt to have a nephrectomy [5,7].

Bladder injuries are reported to be present in 1.6 to 3.6% of abdominal hand gun wound patients [7]. Ureter injuries are an unusual for both blunt and penetrating trauma, but when they occur, they are usually due to hand gun wounds [8]. The majority of patients with ureter injuries have multiple associated intra-abdominal organ injuries, especially small bowel, colon, liver, and iliac vessels. With blunt ureter trauma, multi-organ injuries typically include liver, spleen, and skeletal muscles [8].

In recent literature, renal injuries are managed as conservatively as possible with nephrectomy generally being performed only as a last resort. Conservative management is generally described in guidelines and literature in blunt trauma patients. The practice at the study site has generally been conservative management for penetrating trauma as well unless an injury to the hilum was identified. Indeed, even Grade V kidney injuries are increasingly being managed conservatively [2,6,9]. Lanchon et al. reported successfully using non-operative management in 89% of 124 Grade IV and 52% of 27 Grade V kidney injuries patients [10]. In our patients with kidney injury, if a deadly sign of hypoperfusion was present, the mortality rate for a nephrectomy was 68% vs 31% with no nephrectomy. If no deadly signs were present, the mortality rate for nephrectomy was 12% (2/17). Therefore, if any deadly signs are present, one should avoid a nephrectomy if at all possible. 

With respect to ureteral and bladder trauma, management is dependent upon the clinical setting. Ureteral injuries may be managed with primary repair, stent placement, re-implantation, and/or nephrostomy depending on the location and condition of the ureter and the patient. Uncomplicated extraperitoneal bladder injuries are usually managed with drainage by a urethral catheter [2]. Intraperitoneal bladder injuries or extraperitoneal bladder injury with an adjacent pelvic ring fracture are repaired. In our patients, the isolated bladder injuries were relatively minor, and the mortality rates were low (3/38, 8%). However, patients with an isolated ureteral injury had a higher mortality rate (4/23, 17%, p=0.40).

Just like the seven deadly sins (pride, lust, anger, greed, gluttony, envy, and sloth) described by a Christian monk, Evagrius Ponticus (345-399 A.D.), the seven deadly signs of severe injury and hypoperfusion tend to forecast a bad outcome. In an attempt to combine the major effects of trauma and hypoperfusion, we developed a concept of the seven deadly signs. These include transfusion of 20 or more units of pRBC, severe vascular injury (aorta, IVC, superior mesenteric artery (SMA), or portal vein), Revised Trauma Score 0-4.9, ED systolic BP < 50 mmHg, initial OR systolic BP < 70 mmHg, arterial to end tidal (ET) pCO2 difference (P(a-ET) CO2) greater than 18 mmHg, and lowest OR temperature of less than 33°C, and last OR temperature of less than 34°C. In our patients, three or more of these deadly signs increased mortality rates of patients with GU injuries to greater than 90%.

One of the deadly signs that can be followed closely in the operating threater is the ET pCO2. This parameter is constantly monitored by the anesthesiologist and can be compared to the arterial pCO2 when blood gas analyses are performed. Even if the vital signs and laboratory data are relatively stable, a falling ETpCO2 causing the (P(a-ET) CO2) difference to be more than 10 mmHg should make one suspect increasingly impaired lung perfusion causing an increased pulmonary dead space. In this study, we identified a (P(a-ET) CO2) greater than 18 mmHg to be associated with a mortality rate of 61%. In our previous studies of emergency surgery for trauma, we have noted an increase in mortality rate when an intraoperative (P(a-ET) CO2) is greater than 10 mmHg [11-13]. 

In our patients, the overall mortality rate with GU injuries was significantly higher than that in patients without GU injury (26% (62/242) vs 16% (138/863), p<0.001. This implied that greater depth or force of trauma is required to injure the kidney or ureter. The greatest increase in mortality was in patients having a nephrectomy (55% (29/53)). In addition, the overall increase in the severity of trauma with GU injuries was reflected in the higher ISS (21±11 vs 16±10, p<0.001). DiGiacomo et al. described a trauma cohort of 78 patients with GU injuries who underwent exploratory laparotomy. Of these, 37 patients had a nephrectomy and 16 (43%) died [14]. We also noted that the non-surviving nephrectomy patients had more severe hypotension, higher ISS, more associated injuries and blood loss.

We noted that the overall mortality rate in nephrectomy patients with severe kidney injuries occurred in 55% of patients. When evaluating mortality in nephrectomy patients with deadly signs, two or more deadly signs resulted in a 90% or greater rate of death. We used deadly signs to be reflective of the physiologic stress response from injury. However, we believe that that nephrectomy imparts a greater addition to systemic physiologic shock. Thus, patients with nephrectomy would have a higher rate of mortality versus those patients with increasing numbers of deadly signs as our data demonstrate.

It is well known that blood transfusions are associated with increased risk of multi-system organ failure, systemic inflammatory response syndrome, and higher post-injury infection and mortality rates [15-18]. Of the 72 GU injury patients who received no blood in our study, the infection rate was 14% (10/72). In contrast, in patients receiving blood transfusions, 44% (53/120) developed infection, p<0.001. Indeed, a transfusion of blood was our single most important predictor of post-operative infection.

The main factor involved in increased LOS in our series was the presence of post-injury infection (38±48 vs 9±13, days, p<0.001). Interestingly, the LOS of patients with an isolated bladder injury was similar to those without a GU injury (14±16 vs 15±17, days). The LOS in the patients with ureter or kidney injuries was 23±18 days, p<0.001.

The main limitation of this study is the retrospective cohort design from a single, urban, Level 1 Trauma Center. In this series, the urologic trauma was generally not the only main reason for an emergency laparotomy. Hence, the high mortality and morbidity rates noted here are also a reflection of the severe abdominal injuries. We also recognize the evolution of critical care practice over the course of the study period. We reviewed the ISS and associated mortality within the groups (1980-1989, 1990-1999, and 2000-2010) and found no differences in severity of injury and mortality rates suggesting changes in critical care practice at the study site evolved but did not change mortality rates. In addition, data on urine leaks, development of an urinoma, and creatinine from drained fluids were not recorded.

## Conclusions

In this series of 1105 patients with abdominal trauma requiring an emergency laparotomy, urologic injuries occurring in 242 patients was associated with increased mortality and morbidity rates. The seven deadly signs of hypoperfusion can help determine the severity of injury and prognosis. With a severely injured kidney and any deadly signs of hypoperfusion, special efforts should be made to avoid a nephrectomy.
